# Preparation of Melatonin-Loaded Nanoparticles with Targeting and Sustained Release Function and Their Application in Osteoarthritis

**DOI:** 10.3390/ijms24108740

**Published:** 2023-05-14

**Authors:** Haifeng Liang, Yiran Yan, Wei Sun, Xiaogang Ma, Zhiwen Su, Zhongxun Liu, Yan Chen, Bo Yu

**Affiliations:** 1 Orthopedic and Traumatology Department, Zhujiang Hospital, Southern Medical University, Guangzhou 510282, Chinanealszw@163.com (Z.S.);; 2 Ultrasound Medical Center, Zhujiang Hospital, Southern Medical University, Guangzhou 510282, China

**Keywords:** OA, melatonin, TLR, ROS, PLGA, nanoparticles

## Abstract

(1) The vicious cycle of innate immune response and reactive oxygen species (ROS) generation is an important pathological process of osteoarthritis (OA). Melatonin may be a new hope for the treatment of OA because of its antioxidant capacity. However, the mechanism of melatonin in the treatment of OA is still not completely clear, and the physiological characteristics of articular cartilage make melatonin unable to play a long-term role in OA. (2) The effects of melatonin on ROS and the innate immune response system in OA chondrocytes and the therapeutic effect in vivo were evaluated. Then, a melatonin-loaded nano-delivery system (MT@PLGA-COLBP) was prepared and characterized. Finally, the behavior of MT@PLGA-COLPB in cartilage and the therapeutic effect in OA mice were evaluated. (3) Melatonin can inhibit the activation of the innate immune system by inhibiting the TLR2/4-MyD88-NFκB signal pathway and scavenging ROS, thus improving cartilage matrix metabolism and delaying the progression of OA in vivo. MT@PLGA-COLBP can reach the interior of cartilage and complete the accumulation in OA knee joints. At the same time, it can reduce the number of intra-articular injections and improve the utilization rate of melatonin in vivo. (4) This work provides a new idea for the treatment of osteoarthritis, updates the mechanism of melatonin in the treatment of osteoarthritis, and highlights the application prospect of PLGA@MT-COLBP nanoparticles in preventing OA.

## 1. Introduction

Osteoarthritis (OA) is one of the most common types of arthritis of knee joints. The Global Burden of Disease (GBD) project says there are 303.1 million cases of osteoarthritis of the knee and hip joints worldwide [1]. The crude incidence of osteoarthritis increased by 113.25% between 1990 and 2019 due to the aging of the global population [2]. OA is a complex disease that affects the whole joint. Its manifestations include progressive cartilage invasion, subchondral bone remodeling, and synovitis characterized by diffuse proliferation of lymphocytes [3,4,5]. As the pathogenesis of OA is still unclear, the current treatment of OA is symptomatic treatment, including multidisciplinary non-drug management (exercise, weight loss, and psychological intervention, etc.), drug therapy (non-steroidal anti-inflammatory drugs and steroids), and surgical treatment (arthroscopy and joint replacement) [6]. In addition, drugs such as symptomatic slow-acting drugs for osteoarthritis (SYSADOAs) and prescription-grade crystalline glucosamine sulfate (pCGS) may be effective, but more research is needed [7]. Therefore, finding effective therapeutic targets is an urgent problem to be solved in OA research.

In the course of OA, innate immune response is activated by physical and chemical stimuli, such as mechanical injury and cytokines, through pattern recognition receptors (PRRs), such as toll-like receptor (TLR) [8]. In the early stage of the disease, the inflammatory response caused by the innate immune system can be quickly eliminated by means of cell autophagy, followed by tissue repair. However, when the inflammatory response caused by the damage factors exceeds the body’s ability to repair, the cartilage will receive continuous damage [9]. This persistent chronic inflammation is one of the important causes of OA [8]. The core of innate immune response is the TLR signal, so the TLR and its downstream proteins may be effective targets for the treatment of OA. It has been reported that TLR-mediated activation of the innate immune system is a key step in the development of OA [10]. However, inhibiting the expression of the TLR seems to reduce the ability of chondrocytes to undergo antioxidant stress and autophagy, thus makes them unable to stop the progress of OA [8,11]. On the other hand, excessive production of reactive oxygen species (ROS), that is, oxidative stress imbalance, is one of the important mechanisms of OA disease. ROS mediate a series of adverse reactions in cells, including DNA damage and oxidative damage of proteins and lipids [12,13]. In addition, studies have reported that TLR activation will enrich ECSIT (evolutionarily conserved signaling intermediate in toll pathway) around the mitochondria and ubiquitinate it through TRAF6 (TNF (tumor necrosis factor) receptor associated factor 6), thereby promoting mitochondria to produce ROS, while mitochondrial DNA can also activate and regulate the TLR2/4 pathway through NF-κB (nuclear factor kappa-B), CREB (CAMP response element binding protein), NRF2 (nuclear factor erythroid 2-related factor 2), and IRF (interferon regulatory factor)3 [14,15,16].

The cascade inflammatory reaction mediated by ROS and TLR plays a key role in the course of OA. Therefore, it is a promising treatment for OA to inhibit the production of ROS and the TLR pathway at the same time. Melatonin is an endogenous hormone secreted mainly by the pineal gland, which performs the physiological function of circadian rhythm regulation [17,18]. Furthermore, because of its strong antioxidant capacity, melatonin shows great potential in the treatment of atherosclerosis, osteoporosis, diabetes, cancer, and other diseases [19,20,21]. Previously, melatonin has been reported in the treatment of osteoarthritis [22,23,24], but it has not been found that melatonin can treat osteoarthritis through the TLR2/4 pathway, although some studies have proposed the role of melatonin in regulating the TLR pathway in other chronic inflammatory diseases [25].

**Scheme 1:**
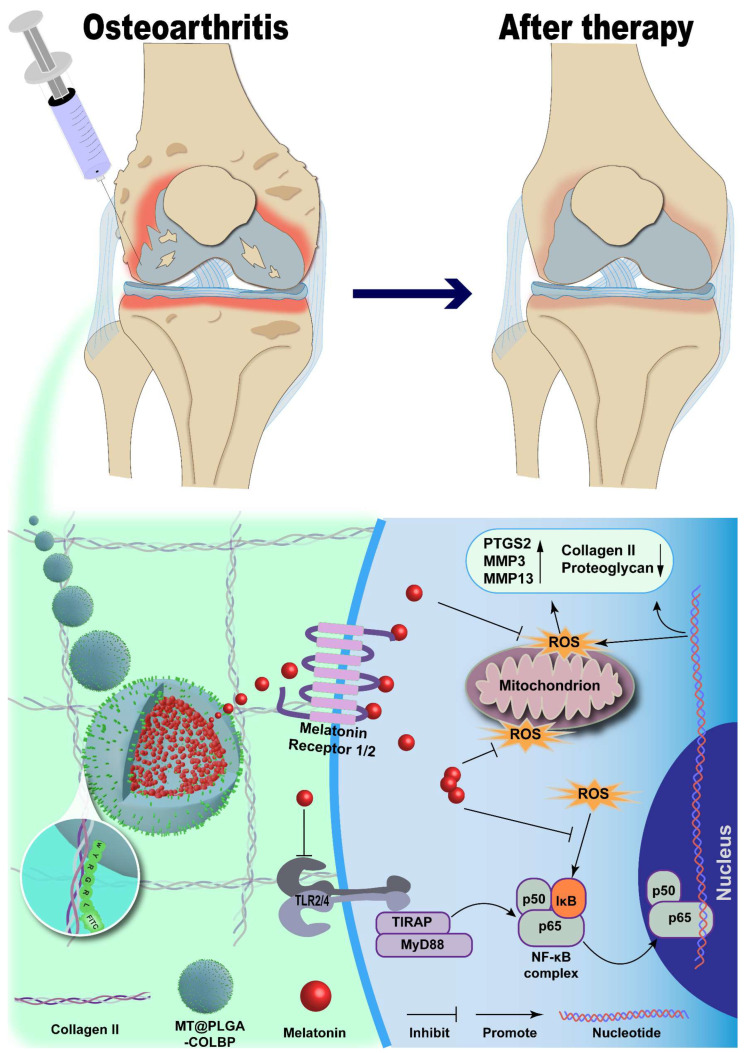
The mechanism of melatonin-loaded nano-delivery system (MT@PLGA-COLBP) with cartilage-targeting effect for osteoarthritis therapy. MT, melatonin; ROS, reactive oxygen species; COLBP, collagen II binding peptide; PTGS2, prostaglandin-endoperoxide synthase 2; MMP3/13, matrix metalloproteinase 3/13. The rising arrow indicates that the protein is up-regulated; the falling arrow indicates that the protein is down-regulated.

Here, we confirmed that melatonin can protect chondrocytes by eliminating ROS and inhibiting the TLR2/4-MyD88-NFκB pathway and prevent the degeneration of knee articular cartilage and remodeling of subchondral bone in early OA mice. However, the traditional route of intra-articular administration leads to the low accumulation and retention of melatonin in articular cartilage. Therefore, in order to improve the utilization rate of melatonin and reduce the frequency of intra-articular drug delivery, there is an urgent need to develop an improved drug delivery strategy (Scheme 1).

Nanopolymers are widely used in the design of nano-delivery systems due to their easy assembly, excellent mechanical properties, high biocompatibility, scalability, good stability, and chemical modification. They can be designed to reduce drug clearance efficiency and improved site-specific delivery, resulting in enhanced therapeutic efficacy and reduced side effects [26,27].

In our study, melatonin was encapsulated in poly(lactic-co-glycolic acid) (PLGA, a polymer material approved by the FDA for use in drug delivery systems [28]) by the water-in-oil method, and then the type II collagen targeting peptide [29] was attached to the surface to prepare a nano-delivery system loaded with melatonin. Nanoparticles can remain stable for at least 21 days and persist in the joint cavity of mice and release melatonin for at least 14 days. Injection frequency of the nano-delivery system was reduced by 75% compared to melatonin-alone injections. The functional release of nanoparticles targeting cartilage and sustained release of melatonin in the articular cavity were realized. Injectable melatonin-loaded PLGA nanoparticles may be a new hope for OA therapy.

## 2. Results

### 2.1. Effects of Melatonin on Proliferation and Function of Chondrocytes

**Figure 1:**
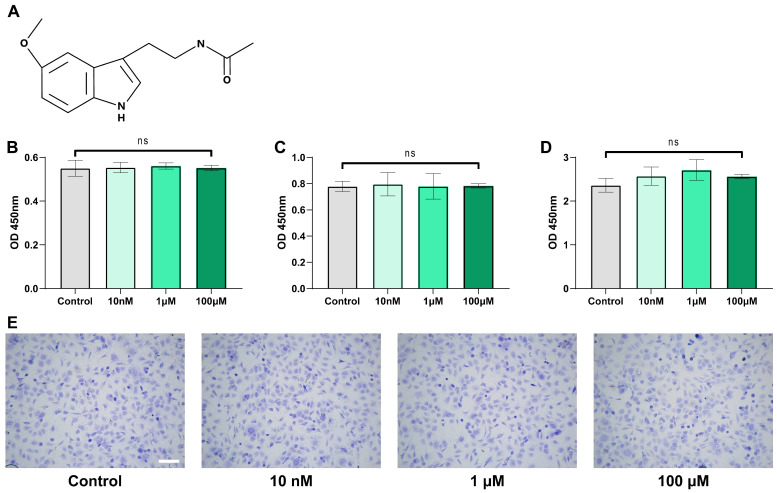
Effects of melatonin on the chondrocyte viability: (**A**) chemical structural formula of melatonin; (**B**–**D**) the OD 450 nm value of chondrocytes exposed to different concentrations of melatonin for 1 (**B**), 4 (**C**), and 7 (**D**) days; (**E**) toluidine blue staining of chondrocytes exposed to different concentrations of melatonin for 72 h; bar = 100 μm. Data are presented as means ± SD; ns means no significance.

The structural formula of melatonin is shown in Figure 1A. In order to study whether melatonin affects the proliferation or function of chondrocytes, we used a CCK8 kit to detect the cell viability after co-culture of chondrocytes with melatonin at different concentrations. The results indicated that the cell viability of chondrocytes exposed to different concentrations of melatonin had no significant difference, whether for 1, 4, or 7 days (Figure 1B–D). In addition, the chondrocytes exposed to different concentrations of melatonin for 72 h were stained with toluidine blue. The results (Figure 1E) showed the morphology of chondrocytes in each group, and there was no significant change in the morphology and staining depth of chondrocytes exposed to different concentrations of melatonin compared with the control group.

Because the biological activity of melatonin is dose-dependent, and based on the above experimental results, it is suggested that 100 μM melatonin is safe for chondrocytes. Therefore, unless otherwise specified, the concentration of melatonin used in subsequent studies is 100 μM.

### 2.2. Melatonin Can Scavenge ROS in Chondrocytes Induced by IL-1β

**Figure 2:**
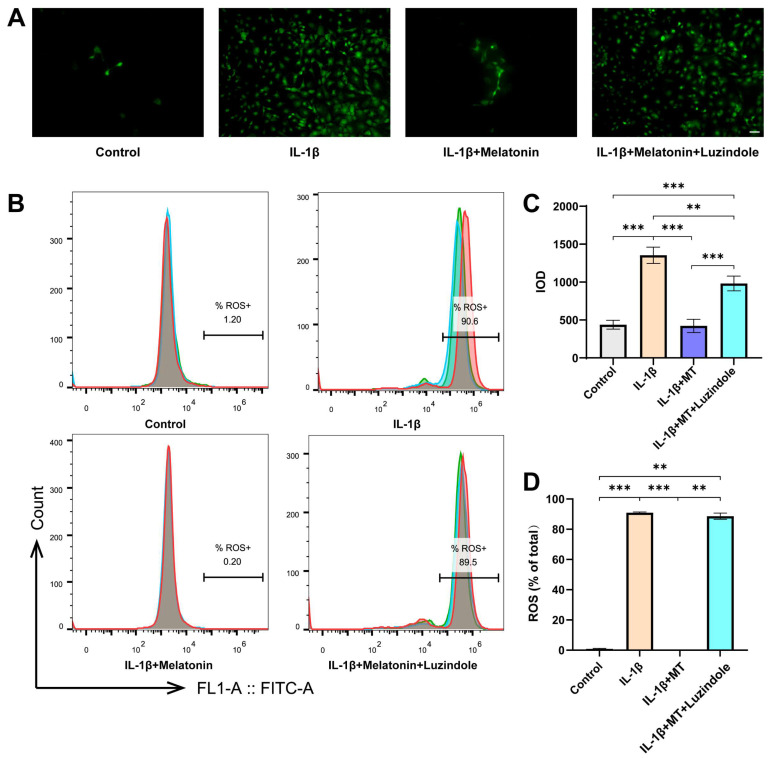
Melatonin scavenges ROS produced by OA cell model: (**A**) representative fluorescent pictures of intracellular ROS produced by chondrocytes after treatment with IL-1β, melatonin, and luzindole, scale bar = 50 μm; (**B**) flow cytometry evaluated the ROS-positive chondrocytes treated with IL-1β, melatonin, and luzindole; three colors represent three independent samples; (**C**) semi-quantitative analysis of integrated optical density (IOD) in (**A**); (**D**) quantitative analysis of ROS-positive cells in (**B**). ROS levels were measured using a DCFH-DA probe. Data are presented as means ± SD: ** *p* < 0.01, *** *p* < 0.001. MT, melatonin.

IL-1β is used to induce primary chondrocytes to become osteoarthritic cell models [30]. We used a 2′,7′-Dichlorodihydrofluorescein diacetate (DCFH-DA) probe to detect the production of ROS (non-specific oxygen free radicals) in chondrocytes of each group. Compared with the control group, a large amount of ROS production was observed in the IL-1β group, which indicated that IL-1β induced oxidative stress injury in chondrocytes. The ROS of the IL-1β + melatonin group decreased significantly, which indicated that the addition of melatonin can counteract ROS induced by IL-1β. On the contrary, ROS increased significantly in the IL-1β + melatonin + luzindole group due to the competitive inhibition of a melatonin receptor (Figure 2A,C). The results of flow cytometry were similar to those observed under the fluorescence microscope; compared with the control group, a large number of positive cells were detected in the IL-1β group, and the proportion of positive cells in the IL-1β + melatonin group decreased significantly, while luzindole could significantly inhibit the protective effect of melatonin (Figure 2B,D).

### 2.3. Melatonin Can Protect Chondrocytes by Inhibiting TLR2/4-MyD88-NFκB Signal Pathway

**Figure 3:**
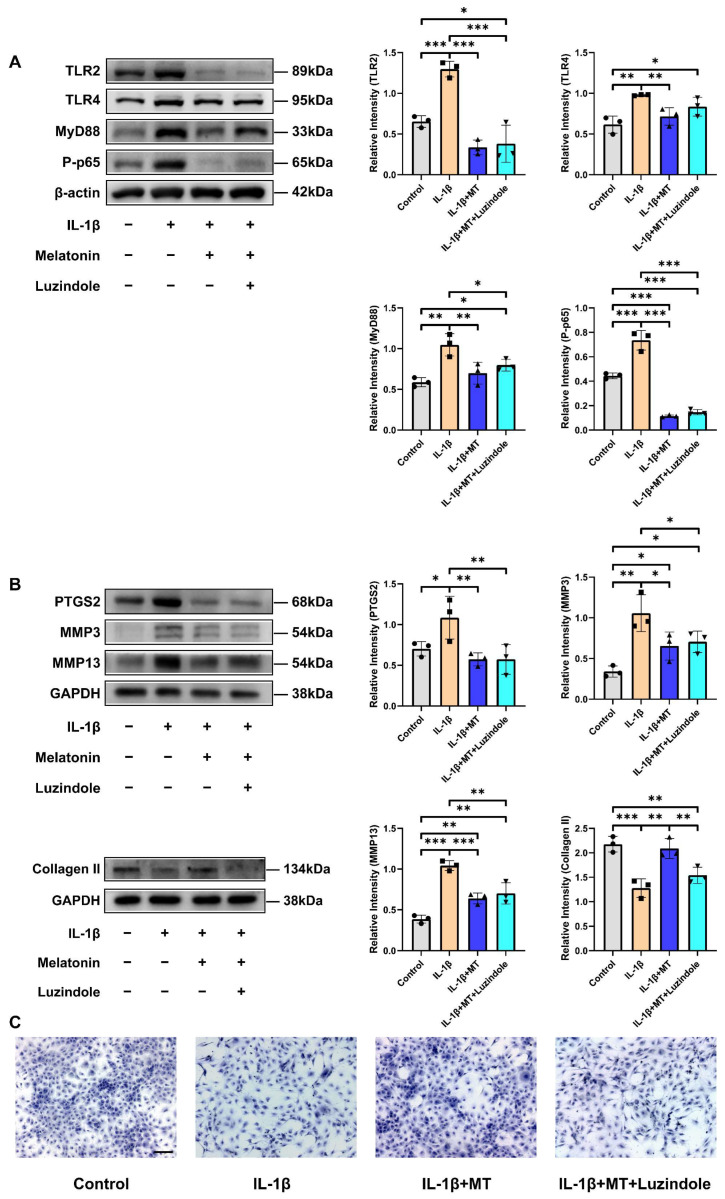
Effects of melatonin on IL-1β-induced TLR pathway and metabolic changes in chondrocytes treated with IL-1β, melatonin, or luzindole for 24 h: Western blot results and band density analysis of (**A**) TLR2, TLR4, MyD88, and P-p65 proteins; (**B**) PTGS2, MMP3, and MMP13 proteins and collagen II proteins of chondrocytes; (**C**) toluidine blue staining of chondrocytes, bar = 100 μm. Data are presented as means ± SD: * *p* < 0.05, ** *p* < 0.01, *** *p* < 0.001. MT, melatonin. Circle, square, triangle and inverted triangle represent independent sample in Control, IL-1β, IL-1β+Melatonin and IL-1β+Melatonin+luzindole group respectively.

To investigate whether melatonin could alter IL-1β-induced activation of innate immune responses and cartilage matrix degradation in chondrocytes, we treated cells with IL-1β, melatonin, and luzindole. After 24 h of exposure, we detected the protein expression of cells. Western blot results showed that the relative expression of TLR2, TLR4, MyD88, and NFκB P-p65 proteins in chondrocytes stimulated by IL-1β increased significantly (Figure 3A). The TLR2/TLR4-MyD88-NFκB signaling pathway in the IL-1β + melatonin group was significantly inhibited. However, luzindole did not reactivate the TLR2/TLR4-MyD88-NFκB signaling pathway in the IL-1β + Melatonin + luzindole group, although the expression of these proteins increased (Figure 3A). Macrophages are one of the most important immune cells in the articular cavity, so when inflammation occurs, the innate immune response of macrophages may also be inhibited by melatonin. As expected, the TLR signaling pathway of macrophages was activated after LPS induction. Melatonin can inhibit this signal pathway (Figure S1). Different from chondrocytes, after exposure to melatonin, the reduction in expression of the activating protein P-p65 of the NFκB pathway in RWA264.7 cells was not as obvious as that in chondrocytes. In other words, the inhibitory effect of melatonin on the NFκB pathway is more obvious in chondrocytes.

In addition, under the action of melatonin, the inflammatory factors (PTGS2) and cartilage matrix degrading proteins (MMP3 and MMP13) of OA chondrocytes in vitro were significantly decreased (Figure 3B), but there was no significant difference in the expression of related proteins between the IL-1β + melatonin group and the IL-1β + melatonin + luzindole group. On the contrary, the expression of type II collagen in OA chondrocytes was significantly increased under the action of melatonin, and the inhibitor luzindole can significantly inhibit the effect of melatonin (Figure 3B). Toluidine blue staining also confirmed the salvage effect of melatonin on type II collagen of chondrocytes and the inhibitory effect of luzindole (Figure 3C). OA chondrocytes are lighter blue and have antennae. Although after melatonin rescue some chondrocytes still appear pale and exhibit antenna growth, most of the cells still maintain the morphology of chondrocytes and the function of secreting collagen.

### 2.4. Therapeutic Effect of Melatonin on Early OA Mice

**Figure 4:**
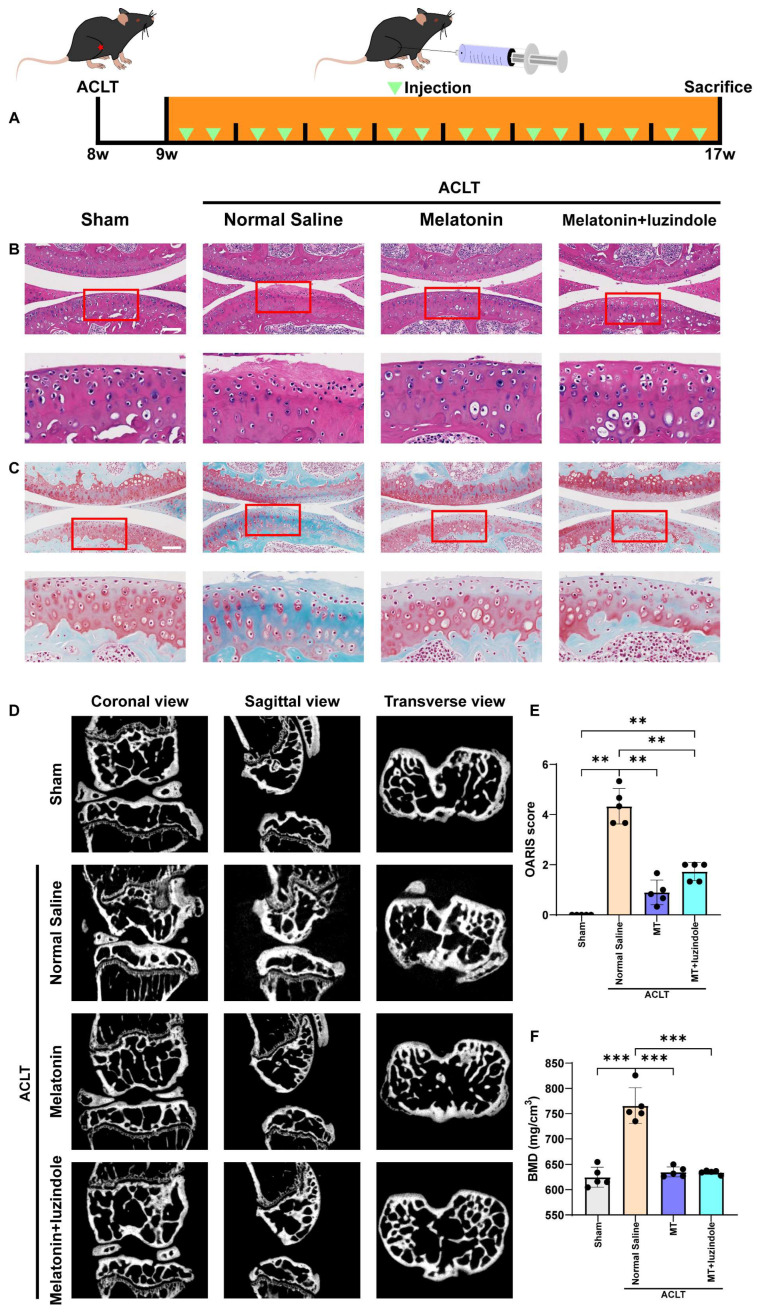
Intra-articular injection of melatonin delays the development of OA in mice. (**A**) Schematic diagram of animal experiment design. C57BL/6 mice underwent anterior cruciate ligament transection at the age of 8 weeks and were treated one week later. The postoperative mice were injected into the knee joint cavity twice a week. The drugs injected were normal saline, melatonin, and melatonin + luzindole. Mice were sacrificed after 8 weeks of treatment. (**B**) Representative picture of knee joint with H&E staining. Bar = 100 μm. (**C**) Representative picture of knee joint with safranin-O fast green staining. Bar = 100 μm. (**D**) Representative diagram of micro-CT of knee joint. (**E**) OARIS score of knee joint based on safranin-O fast green staining. (**F**) Subchondral bone mineral density of tibia based on micro-CT data. Data are presented as means ± SD: ** *p* < 0.01, *** *p* < 0.001. MT, melatonin.

In order to confirm the protective effect of melatonin on cartilage in vivo, the ACLT model was established and treatment was started one week after surgery. Each affected limb received two weekly injections of normal saline, melatonin, or melatonin + luzindole (Figure 4A). Imaging and histological examination were performed after eight weeks of treatment. H&E staining (Figure 4B) and safranin-O fast green staining (Figure 4C) showed the loss of proteoglycan and severe destruction of superficial cartilage in the normal saline group, while the progression of OA was mild in the melatonin group. The competitive inhibition of luzindole on melatonin was also reflected in vivo, with more severe loss of proteoglycan and higher OARSI score (Figure 4E).

On the other hand, the results of a micro-CT showed that the subchondral bone the of normal saline group had significant remodeling, which showed thickening of the bone cortex and an increase in BMD. In contrast, the changes in subchondral bone in the melatonin group and the melatonin + luzindole group were slight, which suggested that melatonin injection can protect subchondral bone from structural changes, while luzindole does not inhibit the protective effect of melatonin on subchondral bone (Figure 4D,F).

### 2.5. Characterization of PLAG@MT-COLBP Nanoparticles

**Figure 5:**
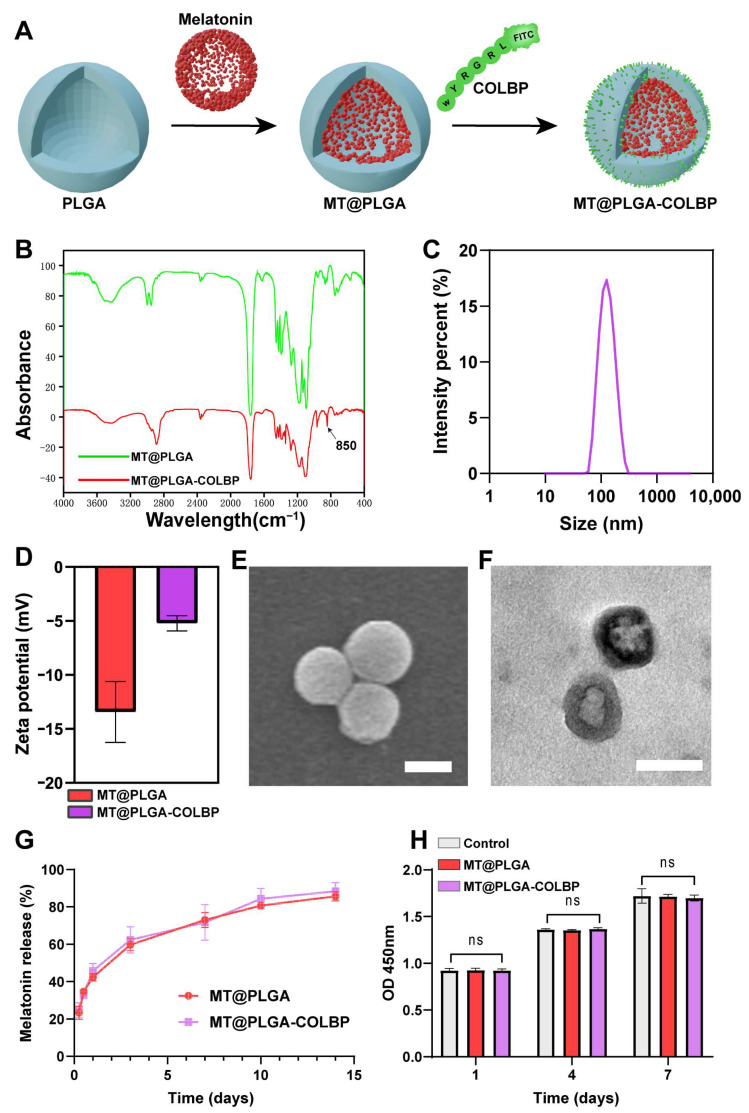
Synthesis and characterization of MT@PLGA-COLBP nanoparticles: (**A**) schematic picture of the synthesis of MT@PLGA and MT@PLGA-COLBP; (**B**) FTIR spectrum of MT@PLGA and MT@PLGA-COLBP; (**C**) size distribution of MT@PLGA-COLBP; (**D**) zeta potential of MT@PLGA and MT@PLGA-COLBP; (**E**) SEM and (**F**) TEM pictures of MT@PLGA-COLBP, scale bar = 50 nm; (**G**) release curve of melatonin in MT@PLGA and MT@PLGA-COLBP; (**H**) the OD 450 nm value of chondrocytes exposed to nanoparticles for 1, 4, and 7 days. Data are presented as means ± SD; ns means no significance. MT, melatonin. COLBP, collagen II binding peptide.

The treatment of melatonin for osteoarthritis is clear, but the maintenance of drug concentration depends on frequent intra-articular injection, which may hinder the application of melatonin. In order to solve this problem, we designed a drug-loaded nanoparticle with PLGA as the main raw material. The internal melatonin is slowly released through the natural degradation of PLGA. At the same time, the polypeptides targeting collagen II were attached to the PLGA nanoparticles to increase the targeting of the nanoparticles and the retention time in the cartilage matrix (Figure 5A). The results of FTIR confirmed the existence of COLBP in the MT@PLGA-COLBP nanoparticles (Figure 5B). The results of the particle size analyzer showed that the hydrated particle size of the MT@PLGA-COLBP is concentrated at 120 nm, and the zeta potential is −5.21 mV (Figure 5C,D). These characteristics can be stable for at least 21 days (Figure S2). The surface morphology and shell–core structure of MT@PLGA-COLBP were obtained by SEM and TEM (Figure 5E,F). Figure 5G shows that MT@PLGA and MT@PLGA-COLBP release melatonin continuously within 14 days, and their sustained release behaviors almost overlap, which proves that the addition of COLBP does not affect the release of melatonin. Finally, we used CCK8 to detect the effect of MT@PLGA and MT@PLGA-COLBP on chondrocytes, and the results showed that the two nanoparticles did not significantly affect the viability and proliferation of chondrocytes (Figure 5H).

### 2.6. Cellular Internalization and Articular Cavity Accumulation of MT@PLGA-COLBP Nanoparticles

**Figure 6:**
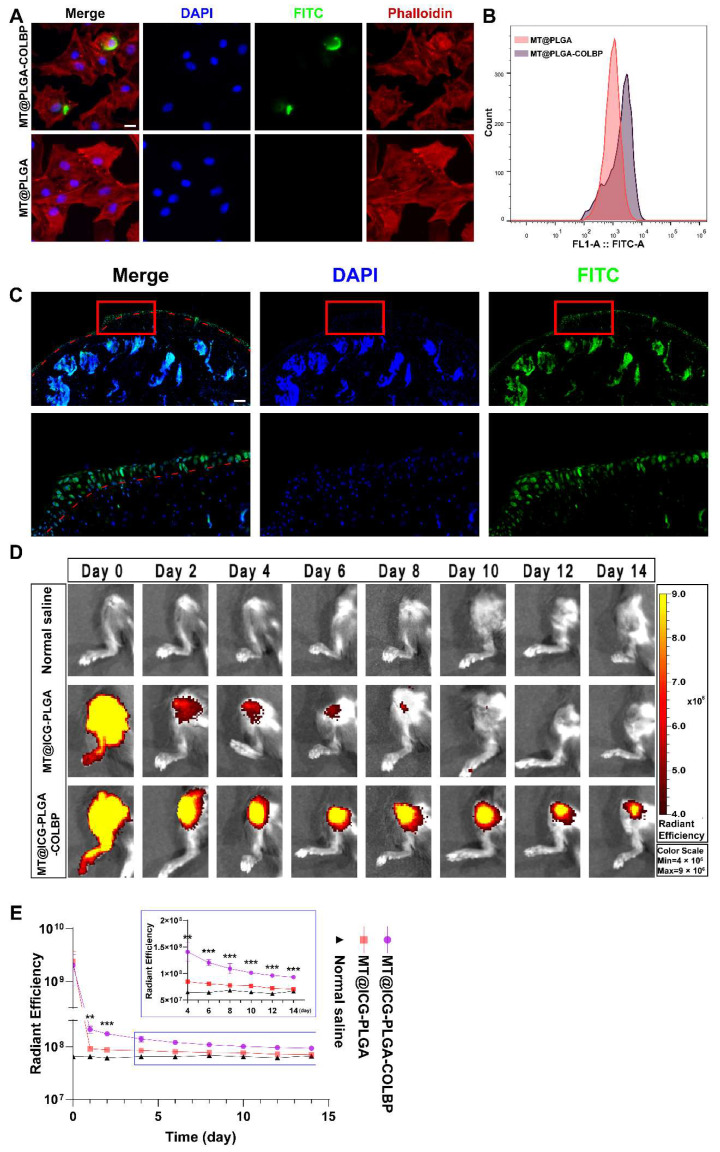
MT@PLGA-COLBP nanoparticles target chondrocytes: (**A**) representative confocal images of MT@PLGA and MT@PLGA-COLBP in chondrocytes, scale bar = 10 μm; (**B**) detection of FITC-labeled COLBP signal in chondrocytes by flow cytometry; (**C**) permeation of MT@PLGA-COLBP in free mouse knee joint, scale bar = 100 μm; (**D**) IVIS pictures of MT@ICG-PLGA and MT@ICG-PLGA-COLBP in the knee joint of ALCT mice; (**E**) analysis of ROI fluorescence intensity in IVIS pictures. Data are presented as means ± SD: ** *p* < 0.01, *** *p* < 0.001. MT, melatonin. COLBP, collagen II binding peptide.

In order to detect the ability of nanoparticles to target chondrocytes in vitro and ex vivo, we co-cultured MT@PLGA and MT@PLGA-COLBP with chondrocytes and free mouse knee joints and detected the fluorescence signals in cells and joints. As shown in Figure 6A,B, after 12 h of co-culture, the fluorescence signal of MT@PLGA-COLBP in chondrocytes can be observed by fluorescence microscope and detected by flow cytometry, which indicates that the nano-delivery system can enter chondrocytes within 12 h. Subsequently, the targeting effect of MT@PLGA-COLBP nanoparticles on the isolated mouse knee joint was also verified. MT@PLGA-COLBP nanoparticles can enter the surface of articular cartilage within 48 h (Figure 6C).

Finally, in order to verify the metabolic cycle of nanoparticles in the articular cavity of living mice, we prepared PLGA nanoparticles modified with ICG: MT@ICG-PLGA and MT@ICG-PLGA-COLBP and injected them into the knee joint cavity of ACLT mice. The non-peptide nanoparticles dissipated on the 10th day after injection, while the signal of the peptide nanoparticles could still be detected on the 14th day (Figure 6D). The fluorescence signals of MT@ICG-PLGA and MT@ICG-PLGA-COLBP showed significant differences from the first day after injection (Figure 6E).

### 2.7. Therapeutic Effect and Biological Safety of PLAG@MT-COLBP Nanoparticles on Early OA Mice

**Figure 7:**
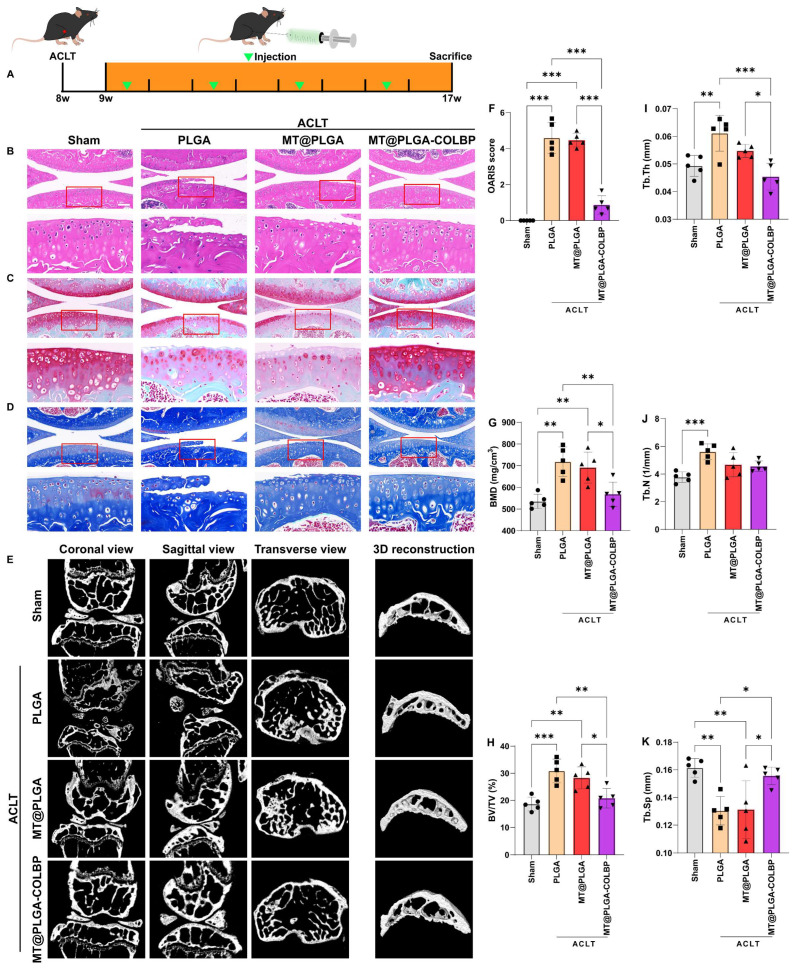
Therapeutic effect of intra-articular injection of MT@PLGA-COLBP nanoparticles on early OA mice. (**A**) Schematic diagram of animal experiment design. C57BL/6 mice underwent ACLT at 8 weeks and were treated one week later. Postoperative mice were injected into the knee joint cavity every two weeks. Mice were sacrificed after 8 weeks of treatment. The injections were PLAG, MT@PLGA, and MT@PLGA-COLBP nanoparticles. (**B**) H&E, (**C**) safranin-O fast green, and (**D**) Masson staining of knee joints of early OA mice. (**E**) Representative diagram and 3D reconstruction diagram of micro-CT of knee joint. (**F**) OARSI score of knee joints of early OA mice based on safranin-O fast green staining. (**G**–**K**) Subchondral bone mineral density (**G**), bone volume/tissue volume (**H**), and thickness (**I**), number (**J**), and separation (**K**) of trabecular bone of tibia based on micro-CT data. Data are presented as means ± SD: * *p* < 0.05, ** *p* < 0.01, *** *p* < 0.001. Circle, square, triangle and inverted triangle represent independent sample in Sham, PLGA, MT@PLGA and MT@PLGA-COLBP group respectively.

In order to study the therapeutic effect of MT@PLGA-COLBP nanoparticles on OA, an early OA mouse model was also established by ACLT surgery. Due to the slow release of melatonin and the targeting of type II collagen by nanoparticles, ACLT mice were injected biweekly into the knee joint and sacrificed eight weeks after treatment (Figure 7A). As shown in Figure 7B–D, a large amount of proteoglycan loss and cartilage erosion occurred in the PLGA group and MT@PLGA group, while the cartilage in the MT@PLGA-COLBP group was well protected. Accordingly, the OARSI scores in the PLGA group and MT@PLGA group were significantly higher than those in the Sham group and the MT@PLGA-COLBP group (Figure 7F). The results of the micro-CT suggested that the subchondral bone structure of the knee joints in the PLGA group was disordered, and new osteophytes were added outside the joints (Figure 7E). The results of BMD and BV/TV showed that the BMD increased in the PLGA group and MT@PLGA group (Figure 7G,H), and the trabeculae in the PLGA group were thicker, more numerous, and had a lower degree of separation (Figure 7I–K). There was no significant difference in related data between the MT@PLGA-COLBP group and the Sham group.

**Figure 8:**
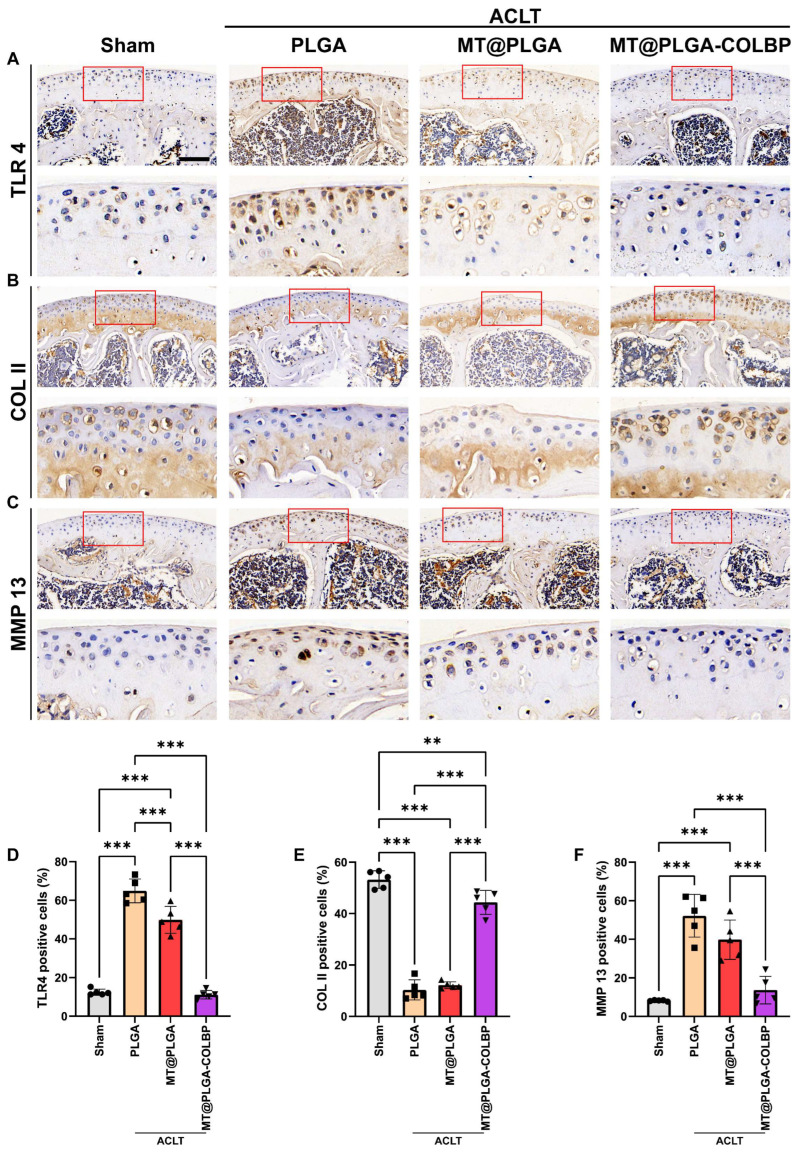
MT@PLGA-COLBP improves the protein expression of immune response and cartilage matrix in vivo. Immunohistochemical staining of TLR4 (**A**), COL II (**B**), and MMP13 (**C**) and analysis of the proportion of positive cells (**D**–**F**). Scale bar = 100 μm. Data are presented as means ± SD: ** *p* < 0.01, *** *p* < 0.001. MT, melatonin. COLBP, collagen II binding peptide. Circle, square, triangle and inverted triangle represent independent sample in Sham, PLGA, MT@PLGA and MT@PLGA-COLBP group respectively.

Finally, we detected the effect of MT@PLGA-COLBP nanoparticles on the protein expression of chondrocytes in vivo by immunohistochemistry. The results showed that early OA induced by ACLT in mice resulted in significant expression of TLR4 but was also inhibited to varying degrees by MT@PLGA and MT@PLGA-COLBP (Figure 8A,D). On the other hand, OA also led to a significant decrease in the expression of collagen II in the PLGA group, and the use of MT@PLGA-COLBP could significantly inhibit the decrease in the expression of collagen II, although it was still significantly decreased compared with the Sham group (Figure 8B,E). Similarly, the in vivo expression of MMP13 was significantly increased in the PLGA group and the MT@PLGA group, and there was no difference between the MT@PLGA-COLBP group and the Sham group (Figure 8C,F). After treatment, there was no significant abnormality in H&E staining of the kidney, lung, spleen, liver, and heart of all mice (Figure S3).

## 3. Discussion

Recently, the therapeutic value of oxidative stress and immune response in OA has been unearthed. Unlike other antioxidants or immunomodulators (such as hyperoside [31], curcumin [32], and astaxanthin [33]), melatonin is an endogenous hormone. Therefore, it is more secure. In fact, melatonin is administered as a dietary supplement or health food rather than a drug in many countries, such as the United States and China [34]. In this study, melatonin in doses of 10 nM–100 μM had no significant effect on the viability and function of chondrocytes. In other studies, the dose of melatonin-treated chondrocytes ranged from 4.3 nM–1 mM, and these reports all concluded that melatonin is safe for chondrocytes [35,36,37,38].

Previous studies have revealed that melatonin can protect cartilage in various ways. For example, melatonin exhibits the following: 1. increases the synthesis and metabolism of chondrocytes by up-regulating TFG-β1 [35]; 2. reduces catabolism by inhibiting the activity of MMPs [39]; 3. protects chondrocytes by activating SIRT1 or inhibiting apoptosis [24]; 4. delays the degeneration of chondrocytes through antioxidant stress [40]. We demonstrated that melatonin can significantly inhibit ROS produced by IL-1β-treated chondrocytes. However, this protective effect was abolished by luzindole, a competitive inhibitor of the melatonin receptor. This shows that the elimination of reactive oxygen species by melatonin is largely dependent on the melatonin receptor. Melatonin has many methods of combating oxidative stress: 1. melatonin and its metabolites can be removed directly by a cascade redox reaction, which is receptor-independent [41]; 2. melatonin increases the activity of antioxidant enzymes, such as glutathione peroxidase (PGx) and superoxide dismutase (SOD), and reduces the activity of inducible nitric oxide synthase (iNOS) through a receptor-dependent pathway [42]; 3. melatonin repairs the impaired mitochondrial function of OA chondrocytes [36]; 4. the metal-chelating activity of melatonin reduces the hydroxyl radicals generated by metal-involved Fenton/Haber–Weiss reactions [43].

As mentioned earlier, the innate immune response mediated by pattern recognition receptors with the TLR as the core is very important during OA. Melatonin is considered to be a natural inhibitor of the TLR [44]. However, the mechanism of melatonin in the treatment of OA through the TLR pathway has been ignored. In our study, the TLR2/4-MyD88-NFκB pathway was significantly expressed in an IL-1β-induced OA cell model. Melatonin showed a significant inhibitory effect on this pathway, and melatonin also up-regulated anabolic proteins and down-regulated catabolic proteins, which further demonstrated the protective effect of melatonin on chondrocytes. Our results show that luzindole has no significant inhibitory effect on melatonin in the TLR pathway of chondrocytes, suggesting that melatonin may affect the TLR pathway of chondrocytes through a receptor-independent pathway. The non-receptor dependence of melatonin on the TLR pathway is similar in RAW264.7. However, in comparison, the promoting effect of melatonin on chondrocyte anabolism is more affected by luzindole, and the mechanism deserves further study. Furthermore, we confirmed the therapeutic effect of melatonin in early OA mice.

Melatonin has a short half-life, about 45 min when taken orally or intravenously [45]. Although intra-articular injection can greatly improve the availability of melatonin, the frequency of injection is still as high as two times per week. In order to improve the utilization rate of melatonin [46] and to reduce the frequency of intra-articular injection to adapt to the long-term treatment of OA, we used MT@PLGA nanoparticles with shell–core structure. After that, the results of FTIR suggest that COLBP was successfully integrated into MT@PLGA nanoparticles. The addition of COLBP can increase the zeta potential of MT@PLGA from −13.43 mV to −5.21 mV, which can reduce the electrostatic repulsion of negative charge in the cartilage matrix and avoid the cytotoxicity of cationic polymer nanoparticles [47]. The particle size of MT@PLGA-COLBP nanoparticles was stable between 100–150 nm in 21 days, which means that melatonin can be stored in the PLGA shell for a long time, which makes up for the short half-life of melatonin. Some scholars believe that only nanoparticles smaller than 60 nm can effectively enter the cartilage matrix [26], but some studies have also proved that 265 nm PLGA nanoparticles can be also transferred to the deep layer of cartilage by a “hitching effect” (nanoparticles are absorbed by synovial macrophages and transported through cell junctions) [27].

Prolonging the retention time of the nano-delivery system in the articular cavity is very important to improve the utilization rate of the delivered drugs [27]. The type II collagen-binding peptide (WYRGRL) used in this study has been shown to specifically bind type II collagen, thereby retaining the drug in the joint [48]. From our experimental results, the nano-delivery system loaded with COLBP remained in the knee joint of ALCT mice for a longer time. When PLGA was degraded, melatonin was released around the chondrocytes and transferred to the cells through melatonin receptors on the cell membrane, or in other ways. On the other hand, similar to the report of Xue et al., the use of type II collagen-binding peptides facilitates the uptake of nanoparticles by chondrocytes, thus promoting the delivery of loaded drugs into cells [49]. The results of Figure 6 confirm the cellular internalization of MT@PLGA-COLBP in vitro and ex vivo.

Although in OA increased vascular and lymphatic permeability due to synovial inflammation may result in faster clearance of nanoparticles [50], in reality, nanoparticles prefer to remain in the OA or aging joint cavity [51]. During OA, the enlargement of cartilage matrix pores caused by the destruction of the type II collagen network [52,53] and the thickening of synovium [54] may be the reasons for the increase in nanoparticle accumulation.

Intra-articular injection of MT@PLGA-COLBP nanoparticles effectively delayed knee joint cavity lesions in early OA mice, including the degradation of the cartilage matrix and changes in subchondral bone. At the same time, MT@PLGA-COLBP nanoparticles also reduced the activation of TLR4 in OA mice and prevented the expression of inflammatory factor MMP13 and the decomposition of collagen II.

There are several limitations to our study. First of all, we did not conduct further research on the failure of melatonin receptor inhibitors, which may involve the receptor-independent pathway of melatonin. Secondly, this study only uses the OA model of ACLT, and does not verify the effect of melatonin/nanoparticles on other OA models. Finally, we lack research on human-derived cells or tissues.

## 4. Materials and Methods

### 4.1. Materials

PLGA, methylene chloride, and ammonium bicarbonate were obtained from Jinan Daigang Biomaterial Co., Ltd. (Jinan, Shandong, China). DSPC and DSPE-PEG2000 were obtained from Avanti Polar Lipids Inc. (Alabaster, AL, USA). PVA, melatonin, and DCFH-DA were obtained from Sigma-Aldrich Inc. (St Louis, MO, USA). FBS and DMEM/F12 were obtained from Gibco Inc. (Billings, MT, USA). All the animals in this study were purchased from JIASIDAJING Biotechnology Co., Ltd. (Guangzhou, Guangdong, China).

### 4.2. Extraction and Culture of Primary Mouse Chondrocytes

Chondrocytes were obtained from the knee joint of seven-day-old C57 mice and cultured in 10% fetal bovine serum (FBS, Gibco, Billings, MT, USA), 5% penicillin streptomycin solution, and 85% Dulbecco’s Modified Eagle Medium/Nutrient Mixture F-12 medium (DMEM/F12, Gibco, Billings, MT, USA). Chondrocytes were incubated at 37 °C in a humidified environment with 5% CO_2_. The culture medium was changed every three days. All chondrocytes were used in the third generation.

### 4.3. Cytotoxicity Test and Toluidine Blue Staining of Chondrocytes

In order to study the effect of melatonin on chondrocytes, referring to the work of Zhou et al. [55], chondrocytes were exposed to 10 nM, 1 μM, and 100 μM melatonin for 1, 4, and 7 days, and a blank control group was set at the same time.

After the above treatment, the chondrocytes were incubated with the medium containing cell count kit-8 (CCK-8, Dojindo, Kumamoto prefecture, Kyushu, Japan) at 37 °C in the dark for 1 h according to the reagent dealer’s working manual, and then the OD value of liquid of each group at 450 nm was measured on the enzyme-labeling instrument (Biotek Synergy H1, Winooski, VT, USA).

Similarly, chondrocytes exposed to 10 nM, 1 μM, and 100 μM melatonin or not for 72 h were fixed with 95% ethanol for 5 min and then stained with toluidine blue staining for 15 min. Finally, the cells were washed with running water and observed under a microscope.

### 4.4. ROS Staining and Flow Cytometry Analysis of Chondrocytes

IL-1β (PeproTech, NJ, USA) is used as an inflammatory inducer for chondrocytes. Luzindole (MedChemExpress, Monmouth Junction, NJ, USA) is a selective melatonin receptor antagonist. Chondrocytes were exposed to IL-1β (10 ng/mL), melatonin (100 μM), or luzindole (10 μM) for 24 h and named as the control group, IL-1β group, IL-1β + melatonin group, and IL-1β + melatonin + luzindole group.

A 2′,7′-Dichlorodihydrofluorescein diacetate (DCFH-D) probe was used to detect intracellular ROS levels (non-specific oxygen free radicals). In short, the adherent chondrocytes were washed by PBS and incubated with a DCFH-DA probe at 37 °C for 30 min. After that, the remaining probes were washed by PBS, and then observed under a fluorescence microscope (Nikon Corporation, Tokyo, Japan). In addition, chondrocyte suspensions (serum-free DMEM/F12) of the above four groups were prepared and incubated with the DCFH-DA probe at 37 °C for 30 min. Intracellular ROS-related fluorescence signals were detected by flow cytometry (Beckman Coulter, Inc., Brea, CA, USA).

### 4.5. Western Blotting Analysis

As mentioned above, chondrocytes were treated with IL-1β (10 ng/mL), melatonin (100 μM), and luzindole (10 μM), and RAW264.7 cells were treated with LPS (10 ng/mL), melatonin (100 μM), and luzindole (10 μM). The two kinds of cells were divided into four groups: control group, IL-1β/LPS group, IL-1β/LPS + melatonin group, and IL-1β/LPS + melatonin + luzindole group.

The total proteins extracted from the cells of each group were added with loading buff and boiled and preserved as protein samples. Protein samples with different molecular weights were separated on sodium dodecyl sulfate polyacrylamide gel electrophoresis (SDS PAGE) gel. Then, the protein on the gel is transferred to the adsorbent membrane carrier under the influence of electric current. The protein on the membrane carrier was blocked by 5% skimmed milk and labeled with specific antibody. Primary antibodies against TLR4 (GB11519, Servicebio, Wuhan, China), TLR2 (#13744, CST, Boston, MA, USA), MyD88 (#4283, CST, Boston, MA, USA), and NF-κB P-p65 (#3033, CST, Boston, MA, USA) were used for chondrocytes and RAW264.7. Primary antibodies against PTGS2 (66351-1-IG, Proteintech, Wuhan, China), MMP13 (18165-1-AP, Proteintech, Wuhan, China), MMP3 (17873-1-AP, Proteintech, Wuhan, China), and COL2 (28459-1-AP, Proteintech, Wuhan, China) were used for chondrocytes. The protein signal carrying the specific antibody was then amplified by the second antibody and visualized in the chemiluminescence instrument (UVITEC, Cambridge, LND, UK). The protein expression was analyzed semi-quantitatively with Image J software (National Institutes of Health, Bethesda, MD, USA, Version 1.8.0).

### 4.6. Preparation and Characterization of MT@PLGA-COLBP Nanoparticles

#### 4.6.1. Preparation of MT@PLGA Nanoparticles and MT@PLGA-COLBP Nanoparticles

First, 5 mg melatonin and 60 mg ammonium bicarbonate were dissolved in 1 mL double-distilled water on ice as an aqueous phase. Then, 100 mg of PLGA, 4 mg of 1,2-distearoyl-sn-glycero-3-phosphocholine (DSPC), and 1 mg of 1,2-distearoyl-sn-glycero-3-phosphocholine-polyethylene glycol 2000 (DSPE-PEG2000) were dissolved in 2 mL of methylene chloride at room temperature as an oil phase. Following this step, 0.4 mL of freshly prepared aqueous phase was added to 2 mL of oil phase. The mixture was phacoemulsified in an ice-water bath by an ultrasonic processor (HUXI, Shanghai, China, 50% amplitude, 30 ms on, 30 ms off) for 4 min. An amount of 10 mL of 3.5% W/V PVA aqueous solution was added to the initial emulsion, and then it was homogenized with a homogenizer (IKA, Germany, 7000 rpm) in an ice bath for 6 min. Then, 16 mL of double-distilled water was added, and the solution was stirred for 4 h at room temperature. The melatonin nanoparticles were then centrifuged at 12,000 rpm for 10 min. The obtained nanoparticles were heavily suspended, rinsed 3 times with double-distilled water, and freeze-dried for 48 h. Finally, the sample was refilled with air to obtain MT@PLGA nanoparticles.

MT@PLGA nanoparticles were incubated with equal mass collb-pep (COLBP, amino acid sequence: WYRGRL [27], labeled with fluorescein isothiocyanate (FITC), prepared by Dia-An, Wuhan, China) at 37 °C for 4 h, and finally stirred at room temperature for 4 h to obtain MT@PLGA-COLBP nanoparticles. MT@PLGA-COLBP nanoparticles also underwent high-speed centrifugation (12,000 rpm for 10 min), double-distilled water cleaning, and freeze-drying (48 h) for standby.

Indocyanine green (ICG)-labeled PLGA was used to prepare MT@ICG-PLGA and MT@ICG-PLGA. In short, the PLGA was replaced with ICG-PLGA (QIYUEBIO, Xi’an, China) before performing the above process.

#### 4.6.2. Characterization of MT@PLGA-COLBP Nanoparticles

The nanoparticles were mixed with potassium bromide at the ratio of 2 to 100, ground, pressed, and then detected by Fourier transform infrared detector (Thermo, Waltham, MA, USA). The hydrated particle size and zeta potential of nanoparticles were obtained by laser particle size analyzer (Malvern, Worcestershire, UK). The freeze-dried nanoparticles were attached to a conductive adhesive, plated with gold, and scanned by a scanning electron microscope (SEM, Phenom G1, Phenom World, Eindhoven, The Netherlands). The ultrasonically dispersed nanoparticle suspension was dropped onto the support film and allowed to stand until the water evaporated completely. The samples were observed by transmission electron microscope (TEM, JEM-1400 Flash, Akishima-shi, Tokyo, Japan) after vacuum coating.

### 4.7. Release Behavior of MT@PLGA-COLBP Nanoparticles

The dosage ratio of dried nanoparticles and PBS soaking solution was 100 mg/50 mL. The sample was put into a shaker with 37 °C. Then, 1 mL of immersion solution was taken at 6 h, 12 h, 24 h, 3 d, 5 d, 7 d, and 2 w, respectively, and 1 mL of PBS solution was added. An ultraviolet spectrophotometer (Thermo, Waltham, MA, USA) was used to determine the release kinetics of melatonin during nanoparticle degradation, including the release amount and release cycle, and the release curve was drawn with degradation time.

### 4.8. Biocompatibility of MT@PLGA-COLBP Nanoparticles

MT@PLGA and MT@PLGA-COLBP nanoparticles (200 μg/mL) were co-incubated with chondrocytes for 1, 4, and 7 days. As described above, a CCK-8 kit was used to detect the OD value of chondrocytes at 450 nm in each group.

### 4.9. Cellular Internalization of MT@PLGA-COLBP Nanoparticles

MT@PLGA and MT@PLGA-COLBP nanoparticles were co-cultured with chondrocytes for 12 h. After the excess nanoparticles were rinsed with PBS, the uptake of the nanoparticles by chondrocytes was observed with confocal microscope through FITC-labeled COLBP. At the same time, the cells of the two groups were digested and resuspended, and the differences in FITC content in the cells of the two groups was detected by flow cytometry to confirm that the nanoparticles were swallowed by chondrocytes.

The culture medium containing MT@PLGA-COLBP nanoparticles was incubated with the free femur of C57BL/6 mice aged 8 weeks for 48 h. The free femur was fixed with 4% paraformaldehyde, decalcified with EDTA decalcifying solution (Solarbio, Beijing, China), dehydrated with sucrose, and underwent frozen section and DAPI staining. Digital Slide Scanners (3DHISTECH, Budapest, Hungary) were used to observe the penetration of nanoparticles in the free femoral head through FITC-labeled COLBP.

### 4.10. Establishment of Early OA Model

All animal experiment procedures were approved by the Institutional Animal Care and Use Committee (IACUC) of Zhujiang Hospital (LAEC-2020-212), Southern Medical University. As we previously reported [30], female C57BL/6 mice aged 8 weeks became the early OA model through anterior cruciate ligament transection (ACLT). In brief, after the mice were anesthetized with isoflurane, a longitudinal incision was made on the lateral side of the mouse knee joint, some of the lateral patellar ligaments were freed, the patella was dislocated, the knee joint was flexed, the anterior cruciate ligament was exposed, the anterior cruciate ligament was transected under direct vision, and then the patella was reset and sewn up layer by layer. In the Sham operation group, only the anterior cruciate ligament was exposed without transection.

Except Sham mice (*n* = 5), all OA model mice were equally divided into the normal saline group, melatonin group, and melatonin + luzindole group (*n* = 5). Mice in the melatonin group and melatonin + luzindole group were injected with 10 μL melatonin (50 mM) and melatonin + luzindole (50 mM + 100 μM) into the knee joint twice a week, and the same amount of normal saline was injected into Sham mice and the normal saline group.

In order to study the accumulation time of nanoparticles in the OA knee joint cavity, 15 OA mice were divided into three groups: normal saline group, MT@ICG-PLGA group, and MT@ICG-PLGA-COLBP group (*n* = 5). One week after operation, 10 μL normal saline, MT@ICG-PLGA suspension (2 mg/mL), and MT@ICG-PLGA-COLBP suspension (2 mg/mL) were injected into the knee joint cavity. The fluorescence signals in the articular cavity of mice were obtained by an In Vivo Imaging System (IVIS Spectrum, PerkinElmer, MA, USA) every two days after injection.

In order to study the therapeutic effect of melatonin-loaded nanoparticles on early OA models, the OA model was randomly divided into the following groups: PLGA group, MT@PLGA group, and MT@PLGA-COLBP (*n* = 5). Groups PLGA, MT@PLGA, and MT@PLGA-COLBP were injected with 10 μL PLGA, MT@PLGA, and MT@PLGA-COLBP (2 mg/mL) every two weeks, respectively, while the Sham group (*n* = 5) was injected with the same amount of normal saline at the same time. All mice were killed after 8 weeks of treatment.

### 4.11. Imaging and Histological Analysis of Early OA Model

The free knee joint was fixed in 4% paraformaldehyde and then micro-CT (ZKKS, Guangzhou, Guangdong, China) scanning was performed. After scanning, the subchondral bone area of the tibia was selected for bone mineral density (BMD) analysis. Three-dimensional reconstruction of subchondral bone for nanoparticles was performed, and the BMD, bone volume/total tissue volume (BV/TV), and trabecular parameters of subchondral bone were analyzed.

The knee joint of mice was fixed in 4% paraformaldehyde and then decalcified in an EDTA decalcifying solution (Solarbio, Beijing, China). Knee joint samples were dehydrated with gradient ethanol, made transparent with xylene, and embedded in paraffin. Following the previous protocols [30], the most representative sections of the knee joint were selected for safranin-O and fast green staining, hematoxylin–eosin (H&E) staining, Masson staining, and immunohistochemical analysis. Among them, primary antibodies against TLR4 (GB11519, Servicebio, Wuhan, China), MMP13 (18165-1-AP, Proteintech, Wuhan, China), and COL2 (28459-1-AP, Proteintech, Wuhan, China) were used for immunohistochemical analysis.

According to Galsson et al. [56], three independent researchers assessed the OA severity in each knee joint, by using the Osteoarthritis Research Society International (OARSI) OA cartilage damage grading system, and the average was taken as the final score of the knee joint.

### 4.12. Statistical Analysis

All data are presented as a mean ± standard deviation. The Student’s *t*-test was used to analyze the differences between the two groups. One-way ANOVA and Tukey’s test were used to compare among groups. A *p*-value < 0.05 was considered significant. At least three independent samples were used for all in vitro experiments and at least five independent samples were used for in vivo experiments.

## 5. Conclusions

Melatonin eliminates ROS in chondrocytes while inhibiting the TLR2/4-MyD88-NFκB signaling pathway, breaking the vicious cycle of ROS innate immune response in OA and improving the synthesis and inhibition of cartilage matrix catabolism. In vivo use of melatonin can effectively delay disease progression in OA mice. Furthermore, we designed a nanoparticle (MT@PLGA-COLBP) with sustained release of melatonin and targeting cartilage. Intra-articular injection of MT@PLGA-COLBP can reduce the number of injections and improve the in vivo availability of melatonin, protecting chondrocytes from ALCT-induced cartilage matrix destruction and subchondral bone changes. The therapeutic effect of melatonin on OA is clear, and its related mechanism and melatonin-based drug delivery system deserve further research and development.

## Data Availability

Not applicable.

## Supplementary Materials

The following supporting information can be downloaded at: https://www.mdpi.com/article/10.3390/ijms24108740/s1.
