# Supplementary material for: Preparation of Melatonin-Loaded Nanoparticles with Targeting and Sustained Release Function and Their Application in Osteoarthritis
Source: Int J Mol Sci. 2023 May 14;24(10):8740. doi: 10.3390/ijms24108740 (PMC10217911; doi:10.3390/ijms24108740)
Supplement: Supplementary file 1 [file ijms-24-08740-s001.zip › ijms-2373711-supplementary.pdf]

Supplementary Materials

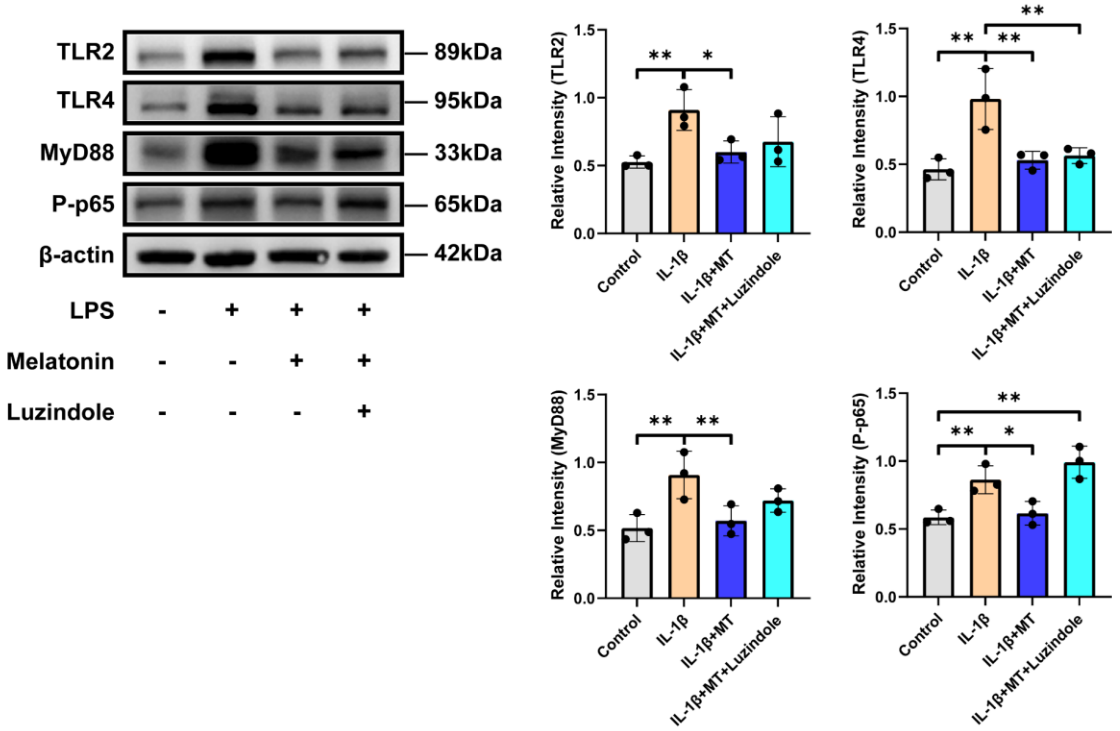

Figure S1. Western blot results and semi-quantitative analysis of TLR2/4-MyD88-NFκB in RAW264.7. Data are presented as means ± SD: * $p < 0.05$, ** $p < 0.01$. MT, melatonin.

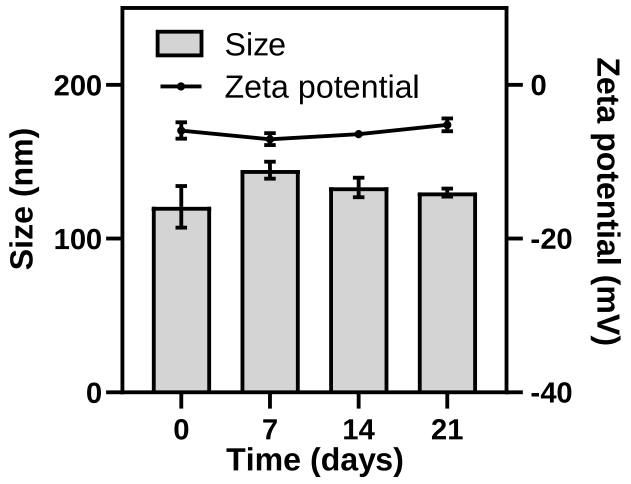

Figure S2. Particle size and zeta potential of MT@PLGA-COLBP nanoparticles at 0-7-14-21 days.

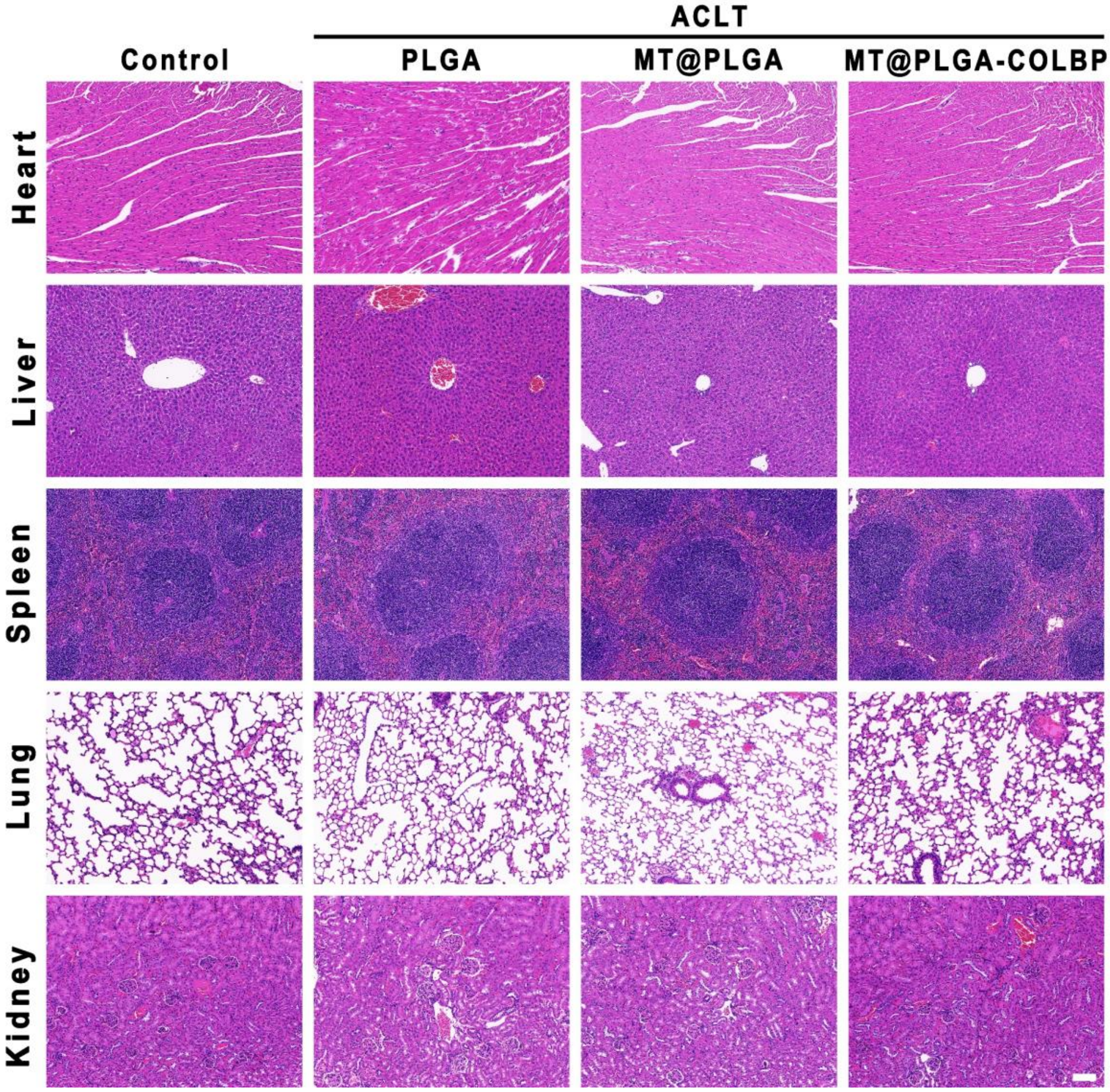

Figure S3. HE staining of mouse heart, liver, spleen, lung and kidney after treatment. Scale bar = 100μm.
